# Pathways to Empowerment as a working method for local social support services

**DOI:** 10.1371/journal.pone.0282470

**Published:** 2023-06-02

**Authors:** Pauline C. van Tol, Irene Jonker, Annabel L. Scheepers, Judith R. L. M. Wolf

**Affiliations:** Department of Primary and Community Care, Impuls—Netherlands Center for Social Care Research, Radboud Institute for Health Sciences, Radboud University Medical Center, Nijmegen, the Netherlands; University of Bucharest, ROMANIA

## Abstract

According to recent legislation, support provided by local authorities in the Netherlands ought to be strengths-based and empower inhabitants to gain control over their lives. This study examined the outcomes, critical elements and working mechanisms of Pathways to Empowerment (PTE), a person-centered, strengths-based intervention, in local social support services provided by a medium size Dutch local authority, from the perspective of citizens needing support. A year after implementation of PTE, semi-structured face-to-face interviews were held with 17 citizens onto their experiences with the provided support with PTE, inquiring their experiences with certain principles of PTE and the changes the support has brought into their lives. The outcomes of support with PTE were: resilience, self-consciousness, positive connections and access to resources and services. According to citizens, ‘being there’, an empowering approach, listening and taking them seriously, focusing on strengths and qualities, working on naturally occurring resources and made-to-measure support is what makes support with PTE work. Working mechanisms connecting the critical elements with the reported outcomes were: building trust and rapport in the client-professional relationship, stimulating trust in and empowerment of self, stimulating social trust and awareness of naturally occurring resources, as well as support, guidance and mediation. The results of this study can help local authorities to better substantiate their choice for applying strengths-based interventions, like PTE, in local social support services.

## Introduction

Since the 1980s, local authorities in Europe have administered several reforms to cope with economic pressures and the increasing urge for more and better service delivery [1]. According to Bovaird [2], the ‘local governance movement’ was born out of a realization that change in urban regeneration in many parts of the USA and Europe became less of a role for the government and more for policy networks and civil society. Characterized as the ‘New Public Management’ (NPM), these reforms aimed to gain more control over policy implementation and its outcomes [3]. At the level of local governments, the local governance movement greatly improved the local government’s autonomy, playing an increasingly important role in the allocation of social resources and the improvement of people’s livelihoods [2].

These reforms taking place throughout Europe also took place in the Dutch welfare domain, in which the shifting expectations of the role of the government was codified in the decentralizations of social care and support provisions. This governmental choice was based on the assumption that local authorities are more familiar with their citizens’ needs and wishes than the national government and know better how to enhance civic participation [4, 5]. Moreover, integral local policies would lead to better provisions of social support tailored to the local situation and would therefore be more cost-effective [6]. As a result of the 2015 Dutch Social Support Act [7], a large part of non-residential care has moved to the local government. Not only is the provision of care seen as a cooperation between various stakeholders such as healthcare providers, insurers and welfare organizations [8], the provision also provides tailored support to each citizen. Moreover, since local authorities are now responsible for fostering self-reliance in their citizens, Dutch local authorities were forced to rethink and adjust their service delivery of social support.

Social support provided by local authorities under the 2015 Dutch Social Support Act [7] is based on the idea that citizens could make more use of their own strengths and are able to fulfill their own needs with the help of fellow citizens. The support is no longer considered to be a citizen’s right, but can only be provided when the capabilities of citizens and their social environment prove to be insufficient to guarantee self-reliance [9]. A perceived lack of social cohesion and mutual engagement in Dutch society were the formal drivers of the Social Support Act, but also rising costs in care in the Netherlands pushed this transformation [10]. The social support itself was also subject to change: it ought to be more personalized, strengths- instead of problem-based, and geared towards empowering citizens to meet their own needs and improve their self-direction, thus taking their own strengths, goals and resources as the starting point of support trajectories. As a result, Dutch local authorities were stimulated to apply strengths-based interventions that would meet these requirements in local social support services, the latter being the first entry point for citizens in need and providing all types of social support, including household care, home adaptations (e.g., stairlifts) and transport (e.g., wheelchairs). People with low education levels, low incomes, a non-Western background, poor health and from a one-parent family appear to make more use of local social support services [10, 11].

In 2019, The Dutch local authority of Nieuwegein, in the West of the Netherlands, with 65,000 citizens and almost 30,000 households, chose to apply *Pathways to Empowerment* (PTE) in their local social support services. However, until now, no local authorities have used PTE in providing social support to citizens in this social domain. This is why the local authority of Nieuwegein not only chose to implement PTE, but also to evaluate the application of PTE from the perspective of users in need of social support. This article reports the findings of this study by focusing on the outcomes, critical elements and working mechanisms of PTE. This study is part of a larger project on the implementation and application of PTE in the local authority of Nieuwegein in the Netherlands.

### Pathways to Empowerment

**Fig 1:**
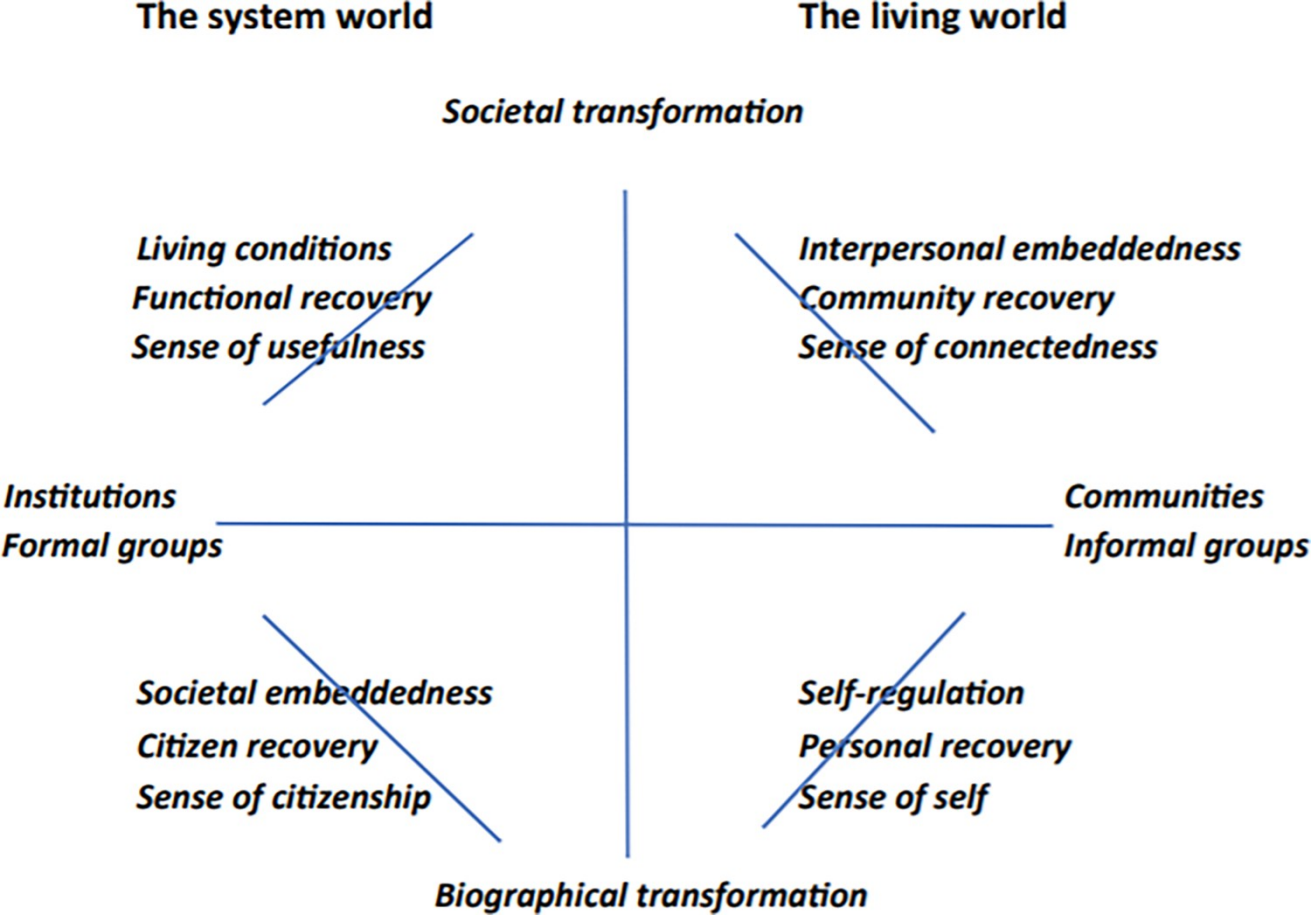
Application of constitutional factors of social quality in Pathways to Empowerment [12].

In the Netherlands, and from 2008, PTE has been implemented in 80 organizations with different target populations in various sectors, such as mental health and addiction care, social care, care for refugees, and forensic care [12]. PTE focuses on individuals who temporarily, or more permanently, experience a loss of control in their lives and have difficulty adequately responding to daily hazards and life challenges. The goal of PTE is to improve the quality of daily life of these individuals by stimulating their personal agency, participation in society, and self-direction in life [12, 13]. To enable growth and recovery in human beings, according to PTE, certain principles could be followed. These ‘strengths principles’ have been adopted from the strengths model of Charles Rapp and Richard Goscha [14], being: 1) Clients with multiple, complex problems are capable of recovering, reclaiming, and transforming their lives 2) The focus is on individual strengths rather than on deficits and problems 3) Clients are the directors of the helping process 4) The client-professional relationship is primary and essential; recovery starts with trust 5) The primary setting for working with individuals is the community; and 6) The community is viewed as an oasis of opportunities and resources [12]. The theoretical framework of PTE originates in the social quality approach (SQA) described by Van der Maesen and Walker [15]. Based on this approach, PTE focuses on four conditions for the quality of daily life of citizens: living conditions (societal-formal), interpersonal embeddedness (societal-informal), societal embeddedness (individual-formal), and self-regulation (individual-informal). Within this framework (Fig 1), ‘living conditions’ refer to the extent to which people have material and immaterial resources over time to be able to live a good life. ‘Interpersonal embeddedness’ describes the level and experience of meaningful, reciprocal positive relationships and a sense of connectedness with other people. ‘Societal embeddedness’, next, is the degree of people’s integration in their communities or society at large and the extent to which people are able to access and make use of it. Finally, ‘self-regulation’ refers to the extent to which people show control of themselves and their lives, being able to alter their internal states, processes, and responses in the anticipation of future goals [12]. Connected to these four ‘constitutional’ factors, PTE distinguishes four types of recovery: a sense of usefulness (living conditions), a sense of connectedness (interpersonal embeddedness), a sense of citizenship (societal embeddedness), and a sense of self (self-regulation) [13]. A sense of usefulness could be accomplished by, for example, organizing the prerequisites and capabilities for a good life, such as housing, income, education and employment. A sense of connectedness, furthermore, could be accomplished by focusing on fostering reciprocity and social recognition, whether in relationships with a partner, children, relatives, friends or other contacts in the community. A sense of citizenship could be enhanced by promoting and sustaining the empowerment of clients as citizens, for example by supporting them to make better use of their basic social rights and the available resources in society. Lastly, a sense of self could be enhanced through supporting and strengthening the personal capabilities and executive functions of clients, thereby stimulating their self-regulation and resilience [12]. Within PTE, these four types of recovery are seen as essential in building agency, participation and self-direction.

No previous studies have investigated strengths-based interventions used by local authorities in social support settings. Past studies on closely related interventions such as the strengths model [14] have been carried out mostly in clinical settings targeting people suffering from chronic psychiatric illnesses. According to these studies, and in comparison to other empowerment-based interventions, the strengths model has been found to lead to a greater reduction in needs for care and less hospitalization [16]. Furthermore, studies have shown the strengths model to lead to a greater reduction in symptoms of mental illness [17], as well as more empowerment and better life satisfaction [18]. For substance abusers, strengths-based care showed a better predictor of involvement in treatment after three months than motivational interviewing or standard care [19]. Better adherence to the care trajectory in case of a strengths-based intervention, compared to care-as-usual, was also found among homeless young adults in the Netherlands [20].

These findings, however, may not be valid for citizens who register for support from local authorities. After all, support trajectories provided by local authorities in the Netherlands are often shorter than within clinical settings [21]. Within these support trajectories, professionals often pay one or several visits to citizens to make an inventory of their needs and requests [21]. After these visits, and if granted, citizens are guided towards appropriate, often more specialized types of care, such as psychiatric help, or they are supported in their request for, for example, a stairlift or a mobility scooter [21].

### Goal and research questions

The current study aimed to extend previous work on strengths-based intervention studies [16–20] to local social support services. In this study, conducted a year after implementation of PTE, three research questions were addressed: 1) What are the outcomes of support with PTE provided by local social support services according to citizens in need of support?; 2) What do citizens perceive as critical elements of the support provided with PTE?; and, 3) What working mechanisms of support with PTE can be identified, based on the perceived outcomes and critical elements? A greater understanding of the value and impact of PTE in short-term support provided by local authorities, from the perspective of its users, could advance the substantiation and application of strengths-based interventions in local social support services in other local authorities, hereby stimulating citizens’ societal participation and self-direction.

### Implementation of PTE

The implementation of PTE in the local authority of Nieuwegein started at the beginning of 2019 with a so-called matching process. In Spring 2019, professionals and citizens of the local authority were consulted–in four working group sessions and one working group session, respectively–in order to explore the fit of PTE with the daily practice of the local social support services and whether any adjustments to PTE, given the new setting and target audience, were necessary. The conclusion was that no major adjustments of PTE were needed. However, an alternative for the strengths assessment (SA), an essential tool of strengths-based interventions, was required to better fit with the short-term support trajectories in the local social support services. It was decided to create a new tool, called the ‘Strengths Compass’. This tool, created by Wolf (JW), developer of PTE, is grounded in the SQA and assesses the strengths, aspirations and resources of citizens, specifically within the four domains of recovery linked to the SQA [15]. It further maps what citizens consider a meaningful life, their wishes and goals for the near future, as well as the actions and those responsible for realizing the goals.

In the Fall of 2019, professionals in Nieuwegein were trained in PTE during a four-day training program, with intervals of two weeks for practice and homework. The training focused on strengths, recovery and hope (Day 1); self-direction, self-regulation, the working relationship, and quality of communication (Day 2); participation, naturally occurring resources, and also setting and achieving goals (Day 3) and the Strengths Compass (Day 4). Training was done by certified PTE trainers linked to the Impuls Academy, being part of the research center of Impuls.

The training of professionals was followed up by six-weekly intervision meetings in small groups, coached by the involved PTE trainers for one year to improve PTE competencies. In this year, the latter also supported the internal PTE coaches, being designated professionals in the teams, to strengthen a correct application of PTE in the daily work of professionals. They were trained by the Impuls Academy to perform the role of coach and received extra hours from the municipal organization to monitor and strengthen the model integrity of PTE as provided in the local social support services. Their tasks were assessing the use of the Strengths Compass by professionals, providing professionals feedback on PTE competencies and offering one-on-one guidance and feedback during their daily work with citizens.

Proper use of PTE was further encouraged in weekly team meetings under the direction of a PTE coach. In this ‘group supervision’, also being an important implementation tool, professionals reflect on their work by making use of the available knowledge and experiences of fellow-colleagues and their input and ideas for working with citizens more effectively.

## Materials and methods

### Local social support services of local authority of Nieuwegein

The study was conducted in the local social support services of the local authority of Nieuwegein, the Netherlands. Different types of support are provided to youth, adults and elderly in different settings (schools, at home) by different types of professionals working in five teams. PTE has been implemented in all five teams: three teams provide support to youth, adults and elderly in specific neighborhoods, one team focuses on support to youth at school, and one team is specialized in providing emergency support in complex situations to citizens with multiple problems. Each team consists of, on average, 14 professionals (in total 72), of which 12 are social workers, one is worker/coach, and one is team leader.

### Design

The current study applied a qualitative design to identify the outcomes, critical elements and working mechanisms of PTE, applied in local social support services of the local authority of Nieuwegein. The study was approved by an accredited Medical Review Ethics Committee region Arnhem-Nijmegen (registration number 2019/5132).

### Procedure

In April 2020, potential participants were first contacted, via telephone, by the professionals of each team. Each team was represented by a random selection of professionals who recruited participants from all teams; recruited numbers of participants were relative to the size of each team. Professionals received instructions from the researcher (PvT) via their internal PTE coach, as well as information via an instruction letter describing the inclusion criteria, contents and duration of the interview that they could use to inform potential participants about the study. If the citizen showed interest in the study and gave permission for the sharing of its contact details, the researcher next contacted the citizen by telephone. The interview, which was held in the period of May to June 2020, took about 45 minutes and was–due to restrictions following the Covid-19 pandemic–held by telephone. All citizens participated in the interview from their home location. At the beginning of each interview, the interviewer (PvT)–who was a PhD student at that time and trained in conducting qualitative interviews–again discussed the conditions for participation in the study. Oral informed consent was then asked, approved by the Institutional Review Board (IRB), which was–similar to the interview as a whole–audio recorded upon permission. After completing the interview, the citizen was sent 10 euros as compensation. Box 1 shows the main questions asked during the semi-structured interview with citizens.

**Box 1. Questions asked during the semi-structured interview with citizens**

1. What was the reason you sought support from the local social support services of Nieuwegein?
2. To what extent did the professional pay attention to your strengths, talents and possibilities during the contact? Was this valuable to you?
3. To what extent did the professional help you be aware of and use resources within your living environment? Was this valuable to you?
4. To what extent did the professional give you hope and perspective in your life? Was this valuable to you?
5. In your life, has somethings changed thanks to the support you received from the local social support services of Nieuwegein? What has changed specifically? And what does this change mean to you?

### Participants

To be eligible to participate, participants had to meet the following inclusion criteria: a) completion of a support trajectory, b) at least one face-to-face contact with a professional, and; c) the latest contact with a professional within a maximum of four months prior to the interview. Of the participating 17 citizens, 14 were female and three were male. Their average age was 40 years (*SD* = 17.45), ranging from 17 to 92 years of age. Of the participants, 70% had an intermediate or lower educational level, whereas 30% had a higher educational level. Most participants were not married (65%) and lived without a partner (53%), although sometimes with children (18%). On average, participants received support during four face-to-face contacts with a professional (*M* = 3.56; *SD* = 2.37), with a minimum of one face-to-face contact and a maximum of eight face-to-face contacts. Next to support in person, participants received support by telephone, text messages and email.

### Analysis

For the analysis, audio recordings were transcribed verbatim. Thematic content analysis was then conducted using the steps as described in the Framework Method by Gale and colleagues [22]. First, two independent researchers (PvT and AS) separately coded the first four interview transcripts line-by-line with the help of ATLAS.ti 8 Windows software, creating codes within the scope of each research question. Next, the two researchers met to compare and agree on a set of codes to be applied to all subsequent transcripts, establishing a ‘working analytical framework’ with defined codes and categories of codes. This working analytical framework, or coding scheme, was then applied by indexing subsequent transcripts using the existing categories and codes and was continuously adapted when new codes and categories would appear. Refinement of the coding scheme–after each set of four interviews–was done after discussion with both the other coder (AS) and the principal researcher (JW). Causal analysis [23] of the descriptive codes was conducted in order to examine the working mechanisms connecting the coded critical elements and coded outcomes.

## Results

The results of this study are reported in terms of outcomes and critical elements of PTE as experienced and reported by citizens needing support from the local social support services. Based on an exploration of the interaction between the perceived outcomes and critical elements, this section will also present the working mechanisms of PTE.

### Perceived outcomes of support with PTE

The outcomes of support with PTE as observed by citizens were: resilience, self-consciousness, positive connections and access to resources and services (Table 1).

**Table 1:** Overview of outcomes of support with Pathways to Empowerment as perceived by citizens needing support from local social support services.

Outcomes	Aspects
Resilience^a^	• Less tension • More well-being • More self-confidence • More hope • More self-direction
Self-consciousness^b^	• More insight into your own problems • More insight into your own qualities • More insight into wishes for the future
Positive connections^c^	• Better family relationships and home atmosphere • Less controlling role as a parent • Stronger informal support network
Access to resources and services^d^	• Access to aids and devices • Access to support services

a In PTE, resilience is mainly influenced by the use of strengths principles, such as expressing belief in the client’s ability to recover, the client being the director in the helping process, the client-professional relationship being seen as primary and essential, as well as exploring client’s strengths and resources and encouraging their use (Wolf, 2016).

b In PTE, self-consciousness is stimulated by exploring and raising awareness of the client’s strengths, qualities and wishes, with the help of a strengths-inventory. Also, belief in the client’s ability to recover and the practice of hope-inducing behavior is important next to the client-professional relationship (Wolf, 2016).

c In PTE, positive connections are stimulated by working in the client’s natural environment and community, gaining more insight into naturally occurring resources and the client’s own network and supporting clients to build or restore relationships (Wolf, 2016).

d In PTE, access to resources and services is stimulated by seeing society as an oasis of opportunities and resources and to encourage clients and their informal and formal care givers to make use of the available opportunities and resources (Wolf, 2016)

### Resilience

Citizens experienced more resilience in terms of reduced tension and higher well-being: ‘*A lot of worry is gone*, *so less stress*. *It also makes you a lot happier*, *because if your child is doing well*, *that means everything’* (F32). Furthermore, citizens felt more self-confident (‘*He didn’t participate in that*, *while he probably would have in the past… Well*, *then he was proud… that also gives him confidence’* [F47]), had better hope for the future (*‘There has been a little more hope that soon there will be support that has not been there all along*’ [F57]) and experienced higher self-direction in life: ‘*I know now there are more options… so I look at more things instead of one thing and if it doesn’t work*, *then I leave it completely and just look at more options’* (F18).

### Self-consciousness

According to citizens, support with PTE further led to higher self-consciousness of their own problems: *‘She did help me to see things more clearly… how quickly I put things*, *problems from the past*, *aside and that these later have a lot of influence on me’* (F18). Also, citizens reported more insight into their own qualities: *‘Another thing she helped me with is to see what I’m good at…’* (F18), as well as wishes for the future: *‘I was able to make my study choice with the help of her’* (F18).

**Positive connections.** Citizens also felt more positive connections within their family: *‘Much tension and worries in our family are gone*, *so that ensures that you just function well together’* (F48). Some parents responded they had less of a controlling role as a parent: ‘*We got a different role again… that we just have a parent-adolescent relationship again*, *instead of having that controlling role with him of “Oh*, *did you do your homework*?*”‘* (F49). Also, relationships within informal support networks improved, according to citizens: *‘Certainly*, *something has changed*. *I have become more open to my friends’* (F17).

### Access to resources and services

Another positive outcome of support with PTE, as perceived by citizens, was access to resources and services. First of all, this applied to aids and devices supporting citizens’ living conditions, such as a mobility scooter and wheelchair: *‘That mobility scooter makes it possible for me to get away for a while and sit in the garden with friends*. *(…) It gives me the opportunity to go away alone for a while’* (M92)*; ‘That wheelchair simply means that I can practice the sport in my own chair and*, *therefore*, *also have more exercise and a more pleasant feeling in it… because it was made just for me’* (M30). Secondly, this applied to access to support services and resources, such as coaching, medication, education, tests, and research. Citizens stated: *‘I now have the life coach*, *that is the most important thing to me’* (F18); *‘Medication for my son*, *that that is going well… education for my son is well organized… that I have a little more help’* (F42)*; ‘Because they offered support*, *he can also go to the [xxx] clinic… who tested him’* (F34).

Most men reported resilience, especially a feeling of self-direction in life, as an important outcome of the support with PTE, whereas most women reported resilience and access to resources and services as an important outcome of support with PTE. Furthermore, most younger respondents made mention of an increase in resilience and positive connections as an important outcome of the support with PTE.

### Critical elements of support with PTE

The analysis of the interviews revealed six critical elements of support with PTE: ‘being there’, empowering approach, listening and taking them seriously, focusing on strengths and qualities, working on naturally occurring resources and made-to-measure support (Table 2).

**Table 2:** Overview of critical elements of support with Pathways to Empowerment as perceived by citizens needing support.

Critical elements	Aspects
Being there	• Taking time for citizens • Being available and accessible • Keeping promises
Empowering approach	• Approaching the citizen as a human being • Showing interest and exploring who the citizen is as a person • Naming what is going well • Offering perspective and suggesting new possibilities
Listening and taking seriously	• Offering a listening ear • Taking citizens seriously • Leaving direction with the citizen • Acknowledging mistakes
Focusing on strengths and qualities	• Exploring and making aware of strengths and qualities • Proposing possibilities and motivating to deploy strengths and qualities
Working on naturally occurring resources	• Making an inventory of support needs from the natural environment • Providing insight into possible resources in the natural environment • Proposing options and mobilizing extra support from the natural environment • Discussing obstacles in the use of resources in the natural environment
Made-to-measure support	• Responding quickly to support requests • Good translation and implementation of needs • Entering into a critical conversation with other parties if needed • Organizing support quickly and effectively • Being clear and transparent

### Being there

Nearly all citizens described that professionals were available and accessible, took plenty of time, and kept promises: *‘He did what he promised… he just always kept his word’* (F36). Another citizen stated: *‘Then she really took plenty of time for us… for all of us*, *including my son’* (F49). Citizens described the professional being available and accessible as follows: ‘*If I had a question*, *I could call or text him and nine times out of ten I got a quick response’* (M30).

### Empowering approach

Citizens felt professionals approached and treated them as a human being (*‘In his eyes*, *his clients are not a file… he talks with feeling’* [M32]*)*, showed interest in who a person really is (‘*Oh you can play football well*, *tell me’* [F47]), mentioned what went well (‘*The way he approached our daughter and spoke to her… how she did it all right’* [F57]) and provided new perspectives: ‘*He was just always positive’* (F36). New perspective was also given by presenting new possibilities to the citizen: ‘*She showed me a door with a lot of options… that there is always a solution for everything… solutions for myself*, *that I can arrange things for myself and that it is not so complicated as I thought’* (F18).

### Listening and taking seriously

Citizens reported that professionals provided a listening ear: *‘She just lets me finish talking and sometimes she repeats*, *in her own words*, *what she has heard from me*, *in order to understand me’* (F24). They felt heard and acknowledged (*‘Giving me a listening ear… which makes me stronger’* [F17]), even more so when the professional left direction with the citizen: ‘*He listens very carefully to what she [daughter] needs… he asks her very clearly*: *“What kind of person do you need*? *Do you want*, *if possible*, *a man or a woman*? *What is important*?*”‘* (F57). This contributed to the ‘building of trust’ in the relationship. Citizens further felt being taken seriously when the professional admitted to having made mistakes: *‘She handled that very well immediately*. *And thus also acknowledged that things had not gone well’* (F49).

### Focusing on strengths and qualities

According to citizens, professionals explored strengths and qualities and made the citizen aware of these by, for example, the use of the Strengths Compass: *‘They had a very large poster and he [son] had to draw his own hand in it first*. *Then*, *he had to write something positive about himself in each finger*, *so his strengths and his good things’* (F48); *‘I do see the benefit of that*. *Then you have very clearly on paper… what your strengths are’* (F17). Furthermore, professionals made compliments to citizens (‘*She has also mentioned*: *“Well*, *you are actually doing this very well*. *You are wonderful parents”*. *That was very nice to hear’* [F49]) and motivated citizens to use their strengths by, for example, stimulating the pursuit of hobbies: *‘Then I tell them I did photography… and then she motivates me very much to continue with it’* (F24).

### Working on naturally occurring resources

Professionals, according to citizens, further explored the citizen’s needs for support from the natural environment (*‘It was looked at and we were asked if we needed it’* [F48]), provided insight into possible sources of support from the natural environment (*‘It has also been made clear what my sources of support are’* [F48]) and encouraged citizens to mobilize sources of support: ‘*Yes*, *in the sense of informal care–my own social circle is actually nil–she has indeed suggested that*’ (F42). Professionals also discussed obstacles in the use of these sources of natural support and how to overcome these: *‘We have given more attention to people who I trust very well*, *but who I don’t want to bother with so much*. *She said it is very important to talk about it… I can see that now’* (F18).

### Made-to-measure support

Citizens mentioned that professionals quickly responded to their request for help (*‘That I called on Wednesday or Thursday… Monday*, *she was at our table’* [F49]). They also felt that professionals met their needs well by showing them the way to receive adequate support: ‘*That it is now also being translated to a party that thinks it can provide good support… we have unfortunately never experienced this before*. *Well*, *that gives me hope and confidence… that it will get on track’* (F57). Furthermore, professionals engaged themselves in a critical conversation with other parties if needed (‘*It is nice if you get the support… that she also says that she talks critically with the school’* [F48]), quickly and effectively organized support (‘*Within a week*, *we also had a list of coaches in the telephone or in the mailbox to see what suited us’* [F49]) and made themselves clear and transparent to the citizen: ‘*He gives me e-mails that he is working on here… or he sends WhatsApp messages or he calls me about “I did this…”*, *so he does give you a constant response*. *Maybe not that day*, *but the day after*’ (M32). Citizens reported that by providing made-to-measure support, professionals provided new perspective, hope and trust.

Most men mentioned an empowering approach as an important element in the provided support with PTE, while most women mentioned the focus on strengths and qualities as being important. Also among younger people this latter element was mentioned most often.

### Working mechanisms of support with PTE

**Fig 2:**
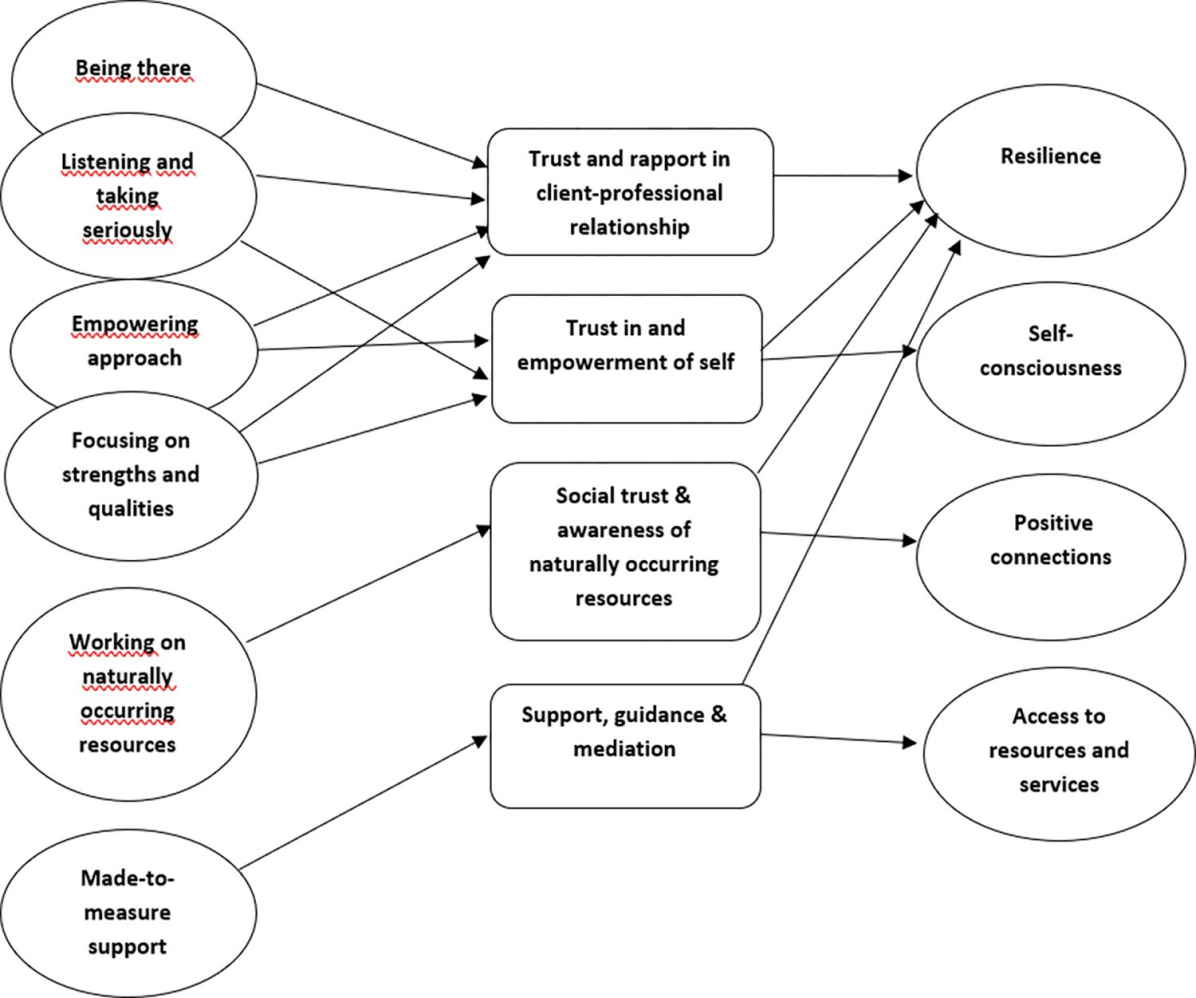
Working mechanisms of support with Pathways to Empowerment in local social support services as perceived by citizens.

Based on an exploration of the interaction between the above-mentioned outcomes and critical elements of support with PTE as perceived by citizens, four mechanisms were identified that make PTE in local social support services work for citizens in need of support: building trust and rapport in the client-professional relationship, stimulating trust in and empowerment of self, stimulating social trust and awareness of naturally occurring resources, and support, guidance and mediation (Fig 2).

### Building trust and rapport in the client-professional relationship

First of all, ‘being there’ for the citizen, providing a listening ear and taking the individual seriously, all add to the building of trust and rapport in the client-professional relationship. In this context, citizens stated: *‘He was simply always available*. *If there is anything*, *I can always text or call him*. *And I just know he’s really trying his best*, *so it makes sense to call him’* (F36)*; ‘Well*, *because he takes it so seriously and knows how to listen very carefully*… *and the fact that you feel clearly heard and… you think*: *“Well*, *now we can build trust again”‘* (F57).

Furthermore, adopting an empowering approach and focusing on strengths and qualities also add to the building of trust and rapport in the client-professional relationship: *‘She was not businesslike*. *It was just a human person*. *(…) It made as if the conversation wasn’t with a professional*, *as if it could have been a friend… it just spoke easier’* (F42)*; ‘His strengths were that he is always cheerful and has humor… So*, *she was really discussing with him things like that*, *so that he felt really comfortable’* (F48). Having trust in the professional, in turn, is important for resilience in terms of hope for a better future, as well as peace of mind: *‘Then it was really like*: *“Oh*, *we have finally been able to clarify what we are looking for*, *for him…*, *and*, *that will be the case now”*. *And*, *not only did we see that with us*, *but also with our son*, *that he was like*: *“Ah*, *it’s alright*, *something is going to happen now”‘* (F49).

### Stimulating trust in and empowerment of self

An empowering approach and the focus on strengths and qualities both add to the stimulation of trust in and empowerment of the self: *‘The way he approached our daughter and spoke to her… where she herself can be quite negative*, *he picks up on the positive things… and with that you try to empower the person’* (F57)*; ‘That gives me a feeling of*: *“Hey*, *you are doing well”… it’s really all positive things she’s actually saying’* (F34). However, also listening to the citizen and taking him seriously add to his self-trust and empowerment: *‘Well*, *that gives him confidence*. *I think she made him feel very understood and he missed that at school’* (F47).

Empowerment of the citizen, in turn, adds to more resilience in terms of, for example, hope for the future (*‘She gave him a new perspective… by emphasizing what he was good at and how he could avoid certain conflicts or anger*, *by dealing with it differently’* [F48]), but also to better dealing with negative emotions: ‘*Well*, *I think he was able to deal with a bit of grief*, *because she approached him as the person he is*… *I think that has taken an enormous burden off his shoulders’* (F48). Also, the stimulation of trust in and empowerment of self leads to more self-consciousness, for example about own qualities: ‘*Then I get to know my characteristics better*, *that I think*: *you can now say you are very insecure*, *but in the meantime you are otherwise very confident’* (F17).

### Stimulating social trust and the awareness of naturally occurring resources

Paying attention to naturally occurring resources in the citizen’s environment creates awareness of possible resources to be used: ‘*Ultimately*, *this came out… in the immediate vicinity… a volunteer organization*. *Yes*. *If she hadn’t mentioned that*, *I wouldn’t have used it’* (F32). Another citizen noticed: *‘That he actually had a lot of friends*, *he found out*, *and all that’* (F47). Furthermore, social trust could be stimulated by being made aware of possible obstacles in relationships and where these come from: *‘I always had the idea that I saddle people with that and that I am a bit of a burden to them… He would always say*: *“Why don’t you do that for yourself to someone else*, *because probably what you give is what you get”*. *That did help me to be more open to my friends’* (F17). Being more aware of resources in one’s natural environment, as well as building more social trust, in turn, results in more positive connections with other people: ‘*Now*, *I also realize that there are other people*. *Yes*, *it is not so bad to talk to other people’* (F18). Also, more resilience in terms of well-being is experienced: *‘This means that once in a while you have contact with other carers… you feel heard’* (F42).

### Support, guidance and mediation

Citizens experienced in almost all cases support, guidance and mediation along the way, which provides relief in the form of hope for the future and the rapid availability of appropriate support: *‘Within a week*, *we had a list of coaches in the phone or in the mailbox to see what suited us*. *That gave us a lot of*: *“Pfff… something happens and help comes… there will be an end to muddling around and getting stuck in support”‘* (F49). Another citizen stated: *‘She arranged things really well*, *so I could just let go of that*. *Because I knew she was going to make it right’* (F32). Mediation, or being willing to critically talk to other parties, further provides a sense of relief and support to citizens: ‘*That she also says that she talks critically with the school… Yes*, *I think that was the thing that helped me the most’* (F48).

Men mentioned building trust and rapport in the client-professional relationship most often, while women most often mentioned stimulating trust in and empowerment of self as an important mechanism in the obtained outcomes of the support with PTE.

## Discussion

This study is the first evaluation of the application of a strengths-based approach within local social support services in the social domain, specifically aiming at exploring the outcomes, critical elements and working mechanisms of the strengths-based intervention, Pathways to Empowerment, from the perspective of the clients. With regard to the outcomes of support with PTE, the findings of this study show that clients perceive more resilience (more well-being and hope for the future), more self-consciousness, more positive connections, and better access to resources and services as positive outcomes of the support provided by professionals applying PTE in local social support services. They consider ‘being there’, showing an empowering approach of the individual, listening and taking the individual seriously, focusing on the individual’s strengths and qualities, working on naturally occurring resources in the environment of the individual and made-to-measure support as critical elements of support with PTE. These critical elements are in alignment with aspects of PTE that could have led to the reported outcomes.

By combining the four outcomes and the six critical elements as perceived by citizens, four working mechanisms of support with PTE emerged. These mechanisms that make PTE work in local social support services are the building of trust and rapport in the client-professional relationship, stimulating trust in and empowerment of self, stimulating social trust and awareness of naturally occurring resources in the individual’s environment, as well as support, guidance and mediation by the professional regarding the request for help.

Working mechanisms and critical elements found within this study strongly overlap with strengths principles from PTE. For example, the mechanism of building trust and rapport in the client-professional relationship relates to the strengths-principle of the client-professional relationship being primary and essential, in which recovery starts with trust [13]. This principle further states that trust is built on mutual respect and authenticity, a supportive client approach, honesty, transparency, optimism, as well as a feeling of equality in the client-professional relationship [13]. These elements could be found in the current results of ‘being there’ for the citizen, listening and taking the citizen seriously and showing an empowering approach. Furthermore, the mechanism of stimulating trust in and empowerment of self, as well as the critical elements of focusing on strengths and qualities and showing an empowering approach–for example by use of the Strengths Compass–overlap with the strengths-principle of the focus being on the individual’s strengths rather than deficits and problems. Also, the mechanism of social trust and awareness of naturally occurring resources, and the critical element of working on naturally occurring resources, relate to the strengths-principle ‘The community is viewed as an oasis of opportunities and resources’. These findings may not be surprising because participants were deliberately asked to reflect on the three strengths principles reflected in the critical elements and mechanisms that were found and discussed here. Results that do not directly pertain to the three strengths principles being studied relate to the mechanism of support, guidance and mediation, and the critical element of made-to-measure support. Providing this type and measure of support lies at the root of PTE, and reflects the strong belief in the recovery potential of the citizen. It also aligns with the strengths principle of ‘Clients with multiple, complex problems are capable of recovering, reclaiming, and transforming their lives’. These results underscore the importance of the underlying principles of PTE in the service to citizens in need of support. Furthermore, the results show that training professionals in PTE principles such as self-direction, the working relationship, the use of naturally occurring resources and the assessment of strengths and qualities directly affects citizens in a positive way.

The results of this study show that the use of PTE in providing generalized social support by local authorities has added value, and can add to the resilience, well-being and informal support of citizens. The outcomes reported by citizens relate to the recovery goals as pursued by PTE. For example, resilience and self-consciousness relate to the recovery goal of ‘a sense of self’ or the constitutional factor of ‘self-regulation’ within the framework of social quality [12, 13]. After all, regaining a ‘sense of self’ means getting to know who one truly is as a person, one’s identity as well as one’s personal values, and from this level of self-consciousness being able to steer one’s own life towards personal goals. If practiced successfully, this leads to more resilience in the form of self-esteem [24, 25].

Furthermore, improved and more positive connections relate to the recovery goal of ‘a sense of connectedness’, which adds to an individual’s social quality via the constitutional factor of interpersonal embeddedness [12, 13]. Regaining a sense of connectedness to other people in the community, as well as within one’s own family, means restoring or renewing the informal relationships with, for example, children and acquaintances, hobby or sports clubs, as well as religious communities and self-help groups [13]. As the results of this study show, awareness of these potential relationships and social trust are important in this domain. This is in alignment with studies showing social trust to be important for a feeling of ‘belonging’ [15, 26]. It further underscores studies emphasizing the importance of being ‘related’ to other human beings for our well-being and self-determination, describing it as a ‘basic psychological need’ [27, 28].

Finally, the perceived outcome of access to resources and services relates to the recovery goal within PTE of ‘a sense of citizenship’ and adds to the individual’s social quality via the constitutional factor of societal embeddedness [12, 13]. Societal embeddedness is regained through better access to social rights and societal institutions. Support with PTE seems to better enable citizens to use or get necessary resources that improve the quality of their daily lives, for example by getting access to education, specialized care and tests, and also aids like a stairlift, a mobility scooter or a wheelchair, which enable them to stay in their home or to engage in activities with friends or to continue doing sports. This relates to the recovery goal of ‘a sense of usefulness’, in which social quality is stimulated by improving own living conditions [12, 13]. Moreover, this is in alignment with what Korevaar and Dröes [29], as well as Donkers [30], mention about ‘functional recovery’, whereby one’s action repertoire is re-evaluated and new competencies are acquired that build trust and lead to more participation in society.

The results of this study are promising, indicating that from the perspective of citizens the support with PTE employed by professionals in local social support services adds to the social quality of citizens’ lives, and empowers them to take control over their lives and fulfill their own needs by making more use of resources in their life world.

### Strengths and limitations

This study has several strengths. First, to our knowledge, this is the first study that has examined the outcomes and the critical elements of a strengths-based methodology–PTE–from the perspective of a group of citizens with a large variety of material and immaterial support needs making use of local social support services. This study has therefore extended past results of the application of strengths-based approaches in clinical settings [16], showing that mechanisms for recovery employed in secondary, specialized care settings could be employed in the social domain, at the entry point providing generalized social care. Another strength of this study is its contribution to the application of strengths-based interventions, such as PTE, in local social support services, providing short-term support to citizens. In alignment with the aims of the local governance movement in Europe [2], the study shows the social support services of a local authority can add to a citizen’s empowerment and access to available resources and services by making use of a strengths-based intervention.

However, although the results clearly show professionals applied trained PTE principles in their daily work with citizens, the current study did not analyze or incorporate the extent to which professionals adhered to the model integrity of PTE. Also, since the local social support services of the municipality of Nieuwegein is the first in which PTE is implemented, the current results cannot be compared to other studies on this strengths-based intervention in the setting of local social support services. Another limitation of this study is that data were collected one year *after* the implementation of PTE within the local authority. Unfortunately, it was not possible to compare these results to data *before* the implementation of PTE. It is possible that the findings could be attributed to the usual support by professionals from this local authority with little added value from PTE itself. Future studies could focus on the longitudinal effects of providing support with PTE over time or on the model fidelity of professionals working with PTE. However, as mentioned here, the critical elements and the outcomes of support with PTE as perceived by citizens strongly relate to the strengths-principles and recovery goals of PTE, whereas citizens also make mention of the added value of the use of the Strengths Compass.

### Implications for practice and policy

Results from the current study provide more insight into citizens’ perception of the added value and outcomes of a strengths-based approach and their related needs. These needs are contained in the found critical elements of PTE, which give professionals direction in the proper use of PTE and how this use could be enhanced. This information is important for professionals providing social support. Outcomes of this study could be used for training purposes, which could further improve this type of short-term support. Furthermore, the results found can better substantiate the choice of local authorities for applying strengths-based interventions, like PTE, in local social support services.

Pathways to Empowerment, a strengths-based approach for use in local social support services to support citizens with various support needs, has added value at both a national and European level. Results underline the shift from problem-based service provision to strengths-based approaches paying more attention to, for example, the strengthening of a citizen’s social resources [2]. Citizens do appreciate how professionals relate to them as human beings, with respect, and how they provide made-to-measure support. They feel heard and taken seriously, and also perceive the support as having a noticeable, positive impact on their lives.

## Conclusions

The strengths-based intervention PTE has positive outcomes for the resilience, self-consciousness, social connections and access to resources and services of citizens. Critical elements playing a role in these outcomes are ‘being there’ for the citizen, showing an empowering approach of the individual, listening and taking the individual seriously, focusing on the individual’s strengths and qualities, working on naturally occurring resources in the environment of the individual and providing made-to-measure support. These elements are important to implement and enhance local social support services on a wider scale so as to stimulate the transition from problem-based to strengths-based service provisions and to strengthen a citizen’s participation and self-direction in life.
